# A rare case report of acute severe hypokalemia induced by monosialotetrahexosylganglioside therapy

**DOI:** 10.1097/MD.0000000000045275

**Published:** 2025-10-31

**Authors:** Xiang Zheng, Jvping He

**Affiliations:** a Department of Pharmacy, Dongyang People’s Hospital, Zhejiang, China; b Department of Neurology, Dongyang People’s Hospital, Zhejiang, China.

**Keywords:** absent tendon reflexes, case report, hypokalemia, weakness

## Abstract

**Rationale:**

Monosialotetrahexosylganglioside (GM1), an exogenous ganglioside, is widely used as an adjuvant in treating cerebrovascular diseases and Parkinson disease. While its association with Guillain–Barré syndrome is well-established, GM1-induced hypokalemia presenting-with overlapping clinical features such as muscle weakness-has not been previously reported. Severe hypokalemia can cause neurological deficits and electrocardiographic abnormalities, emphasizing the need to recognize this potential adverse effect.

**Patient concerns:**

A 68-year-old male with parkinsonism developed severe lower extremity weakness (inability to walk unassisted) and absent tendon reflexes 6 days after initiating GM1 therapy.

**Diagnoses:**

Laboratory testing confirmed severe hypokalemia (serum potassium: 2.05 mmol/L). The Naranjo Adverse Drug Reaction Probability Scale scored 6 for GM1, indicating GM1 as the more probable cause.

**Interventions:**

Immediate potassium supplementation was administered, and GM1 was discontinued.

**Outcomes:**

Following interventions, the patient’s muscle strength and tendon reflexes improved significantly (serum potassium: 3.99 mmol/L), with resolution of hypokalemia.

**Lessons:**

Older adults with comorbidities are vulnerable to GM1-associated hypokalemia. Clinicians should monitor potassium levels during GM1 therapy, and further research into underlying mechanisms is warranted.

## 1. Introduction

Monosialotetrahexosylganglioside (GM1), an exogenous ganglioside widely used in clinical practice in China, is composed of sialic oligosaccharides and ceramide.^[1]^ Due to its lipophilic nature, GM1 exhibits high affinity for neuronal membranes, where it modulates membrane functionality and promotes the remodeling and regeneration of damaged neural cells. Clinically, it is frequently employed as an adjuvant therapeutic agent in conditions such as acute cerebral infarction, traumatic brain injury, and neurodegenerative disorders.^[2,3]^ The incidence of cerebrovascular accidents has increased in recent years, with reported mortality and disability rates of 1.4‰ (2020) and 22.2% in (2019), respectively.^[4]^

Despite its therapeutic benefits, GM1 administration has been associated with various adverse effects, including systemic, dermatological, musculoskeletal, visual, gastrointestinal, and urinary complications.^[5]^ Among these, Guillain–Barré syndrome (GBS) is a particularly severe and well-documented adverse effect.^[6]^ GM1-related GBS (GRD-GBS) typically occurs approximately 2 weeks after the initiation of treatment.^[6]^ The primary clinical manifestations of GBS include muscle weakness and diminished or absent deep tendon reflexes.^[7]^

Electrolyte disturbances, particularly severe hypokalemia, can similarly induce alterations in muscle strength and physical signs. The severity of hypokalemia-related clinical symptoms is generally proportional to the degree and duration of the electrolyte disturbance.^[8]^ The relationship between GM1 use and severe hypokalemia (< 2.5mEq/L) remains uncommon and poorly documented in the literature. Moreover, this adverse effect is not explicitly mentioned in the prescription information of the drug. Emerging preclinical evidence suggests GM1 may disrupt potassium homeostasis through effects on ion channels and transporters, providing a theoretical basis for this association.^[9,10]^ This case report aims to emphasize the importance of monitoring electrolyte levels in patients receiving GM1 therapy to mitigate potential risks, while acknowledging that the Naranjo Adverse Drug Reaction Probability Scale (NADRPS) score for GM1 in this case is 6-a score that, indicates a “probable association” rather than an absolute causal relationship, which represents a key limitation in definitive causal inference.

## 2. Patient information

A 68-year-old married male with suspected parkinsonism was admitted to the neurology ward due to recurrent falls and progressive memory deterioration. One day prior to admission, the patient experienced a fall to his left side, resulting in a bruised cheekbone, without loss of consciousness. Physical examination on admission revealed bradykinesia and resting tremor, with normal tendon reflexes.

His medical history included cerebellar infarction, benign prostatic hyperplasia, hypertension, and parkinsonism. Upon admission (day 1), his treatment regimen consists of clopidogrel (75 mg daily), atorvastatin (20 mg nightly), finasteride (5 mg daily), tamsulosin hydrochloride sustained-release capsules (0.2 mg nightly), amlodipine besilate (5 mg daily), and GM1 (40 mg intravenous infusion daily, initiated on day 1). on day 2, Irbesartan (150 mg daily) was added to address uncontrolled hypertension.

On day 6, a 24-hour Holter electrocardiogram detected suspicious U-wave changes and simulated ST-T segment changes. The patient remained asymptomatic until 00:15 on day 6 (November 4), when he developed limb weakness and inability to walk independently. Physical examination revealed reduced muscle strength (left upper limb: grade 4, right upper limb: grade 5-, left lower limb: grade 3, right lower limb: grade 2) and absent tendon reflexes. Laboratory testing confirmed severe hypokalemia (serum potassium: 2.05 mmol/L; baseline serum potassium on admission was within normal limits). Emergency potassium supplementation (Potassium Chloride 1 g intravenous infusion) was administered, and GM1 was discontinued on day 6. Electromyography showed axonal damage in both lower limbs; this finding is consistent with hypokalemic myopathy, as severe hypokalemia is known to impair neuromuscular transmission and induce reversible axonal dysfunction.^[11]^ Endocrine evaluations yielded normal results (serum cortisol: 9.97 μg/dL [8:00], 6.51 μg/dL [16:00], 10.80 μg/dL [24:00]; free T3: 4.28 pmol/L [normal: 3.1–6.8 pmol/L]; free T4: 17.70 pmol/L [normal: 12.0–22.0 pmol/L]; TSH: 4.11 μIU/mL [normal: 0.27–4.2 μIU/mL]; aldosterone: 21.26 pg/mL [normal: 26.89–225.12 pg/mL]), ruling out endocrine disorders (e.g., hyperthyroidism, primary hyperaldosteronism, Cushing syndrome) as causes of hypokalemia. Brain MRI and bilateral adrenal gland imaging, and renal function tests were unremarkable.

**Figure 1.**
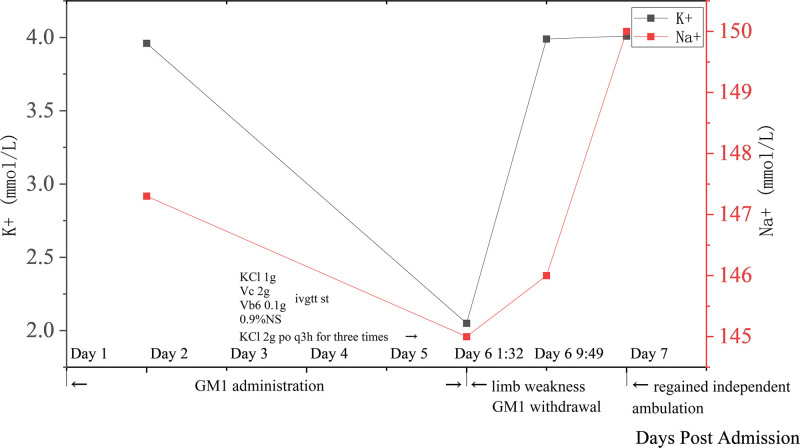
Clinical course, potassium and sodium levels in the case: dynamic changes in serum potassium () and sodium (● ) levels during hospitalization. Arrows denote therapeutic interventions.

By day 7, the patient reported significant improvement in lower limb muscle strength and regained independent ambulation. Muscle strength normalized to grade 5 in both upper limbs and grade 4 in both lower limbs, with tendon reflexes was +. Serum potassium levels normalized to 3.99 mmol/L after the single intravenous potassium dose and remained within the normal range thereafter. The trend of patients’ serum potassium levels and clinical course during hospitalization are shown in Figure. 1 .

## 3. Discussion

Following GM1 discontinuation and potassium supplementation, the patient’s serum potassium levels normalized with with concurrent improvement in muscle weakness, exhibiting a clear temporal relationship: symptoms onset 6 days after GM1 initiation (day 6), serum potassium decline from baseline (3.96 mmol/L on day 2) to severe hypokalemia (2.05 mmol/L on day 6 1:32), rapid potassium recovery (3.99 mmol/L on day 6 9:49, 4.01 mmol/L on day 7) and symptom improvement within 1–4 days of GM1 withdrawal and potassium supplementation. This sequence is explicitly plotted in Figure 1 for clarity.

This clinical improvement is attributed to the reversal of hypokalemia-induced peripheral nerve dysfunction. Hypokalemia typically arises from 3 primary mechanisms: reduced dietary intake, enhanced intracellular potassium shifts, or increased potassium loss (via the kidneys, gastrointestinal tract, or sweat).^[12]^ Our patient’s normal thyroid function (free T3, free T4, TSH) and aldosterone/cortisol levels ruled out primary hyperaldosteronism or Cushing syndrome. Regarding potential drug-drug interactions: the patient’s medication regimen included amlodipine (calcium channel blocker), irbesartan (angiotensin II receptor blocker), clopidogrel (antiplatelet), atorvastatin (statin), finasteride (5α-reductase inhibitor), and tamsulosin (α1-adrenergic blocker). A review of pharmacovigilance data and drug interaction databases (e.g., Lexicomp, Pubmed) showed no established interactions between these agents that would induce severe hypokalemia. In this case, the patient denied appetite disturbances, and no evidence of renal disease, diabetes, or gastrointestinal loss was identified, eliminating these as potential causes.

GM1 is recognized for its neuroprotective properties in Parkinson disease and central nervous system injuries,^[5,13]^ but is limited by serious adverse effects, particularly immune-mediated neurological disorders such as GBS and multifocal motor neuropathy.^[14–16]^ Electromyographic findings in such cases often indicate peripheral nerve demyelination or axonal degeneration.^[17]^ Notably, our patient’s axonal changes, detected only after the onset of severe hypokalemia and in the absence of baseline or follow-up EMG data, cannot be definitively attributed to GM1-induced neuropathy or preexisting neurological conditions; however, their temporal alignment with electrolyte disturbance and clinical recovery strongly suggests a reversible, hypokalemia-mediated process. Additionally, GM1 administration may stimulate the production of anti-ganglioside antibodies, which along with complement-mediated complexes, can target axonal membranes, impair sodium channels, reduce sodium influx, and inhibit action potential generation.^[18]^ Importantly, we did not measure anti-ganglioside antibody levels or directly assess complement activation in this patient; thus, this immune-related mechanism remains speculative and unsupported by direct clinical evidence from the current case. Potential mechanisms by which GM1 may lead to decreased serum potassium levels can be elaborated as follows.^[9,10]^ Enhanced Na⁺, K⁺-ATPase activity promoting intracellular K⁺ sequestration. Potassium channels activation and redistribution. Activated K⁺ channels and in conjunction with the enhanced Na⁺, K⁺-ATPase activity, this effluxed K⁺ is likely to be further taken up by cells, collectively contributing to a potential reduction in serum potassium levels. While preclinical literature provides theoretical support for these ion channel- and immune-related mechanisms linking GM1 to potassium homeostasis disruption, the absence of direct measurements in our patient means these remain hypothetical and require further validation.

GM1 received a NADRPS score of 6 as detailed in Table 1. The adverse aligns with the 6-day onset of symptoms-consistent with the timeline of drug steady-state achievement (t1/2:~1 day),^[19]^ strengthening its association with the observed hypokalemia.

**Table 1:** The assessment of suspected medications by Naranjo Scale.

Question	GM1
Are there previous conclusive reports on this reaction?	0
Did the adverse event appear after the suspected drug was administered?	+2
Did the adverse reaction improve when the drug was discontinued or a specifc antagonist was administered?	+1
Did the adverse event reappear when the drug was re-administered?	0
Are there alternative causes (other than the drug) that could on their own have caused the reaction?	+2
Did the reaction reappear when a placebo was given?	0
Was the drug detected in blood (or other fuids) in concentrations known to be toxic?	0
Was the reaction more severe when the dose was increased or less severe when the dose was decreased?	0
Did the patient have a similar reaction to the same or similar drugs in any previous exposure?	0
Was the adverse event confrmed by any objective evidence?	+1
Total	6

GM1 = monosialotetrahexosylganglioside.

The patient reported strict adherence to medical advice regarding potassium supplementation and serum potassium monitoring throughout treatment. He noted appreciation for the professional care received during symptom resolution, which increased his awareness of abnormal health changes.

Close monitoring of electrolyte levels is advisable during GM1 therapy. To our knowledge, this report is the first to describe severe hypokalemia associated with GM1 use, with a NADRPS score of 6 indicating a probable association. However, given the inherent limitations of case reports (e.g., single-patient data, inability to control for all confounders), and the lack of direct evidence for the proposed ion channel- and immune-related mechanisms in this case, definitive confirmation of causality requires further research. This should include in vitro studies examining GM1’s effects on potassium transporters, larger retrospective or prospective clinical cohorts, and ideally controlled studies-to validate these findings and clarify the incidence and clinical significance of this potential adverse effect.

## Author contributions

**Conceptualization:** Xiang Zheng.

**Investigation:** Xiang Zheng, Jvping He.

**Supervision:** Jvping He.

**Writing – original draft:** Xiang Zheng.

**Writing – review & editing:** Jvping He.
